# High-density genetic map and genome-wide association studies of aesthetic traits in *Phalaenopsis* orchids

**DOI:** 10.1038/s41598-022-07318-w

**Published:** 2022-02-28

**Authors:** Chia-Chi Hsu, Shu-Yun Chen, Shang-Yi Chiu, Cheng-Yuan Lai, Pei-Han Lai, Tariq Shehzad, Wen-Luan Wu, Wen-Huei Chen, Andrew H. Paterson, Hong-Hwa Chen

**Affiliations:** 1 Department of Life Sciences, National Cheng Kung University, Tainan, 701 Taiwan; 2 Department of Plant Biology, Franklin College of Arts and Sciences, University of Georgia, Athens, GA 30602 USA; 3 Orchid Research and Development Center, National Cheng Kung University, Tainan, 701 Taiwan; 4 Present Address: CH Biotech R&D Co., Ltd, 89, Wen-Hsien Rd, Nantou County, 54041 Taiwan; 5 Present Address: Department of Agronomy, College of Agriculture and Natural Resources, National Chung-Hsin University, Taichung, 402 Taiwan

**Keywords:** Functional genomics, Genetic association study, Genetic markers, Genomics, Genotype, Plant breeding, Plant genetics, Population genetics, Quantitative trait

## Abstract

*Phalaenopsis* spp. represent the most popular orchids worldwide. Both *P. equestris* and *P. aphrodite* are the two important breeding parents with the whole genome sequence available. However, marker–trait association is rarely used for floral traits in *Phalaenopsis* breeding. Here, we analyzed markers associated with aesthetic traits of *Phalaenopsis* orchids by using genome-wide association study (GWAS) with the F1 population *P.* Intermedia of 117 progenies derived from the cross between *P. aphrodite* and *P. equestris*. A total of 113,517 single nucleotide polymorphisms (SNPs) were identified in *P.* Intermedia by using genotyping-by-sequencing with the combination of two different restriction enzyme pairs, *Hinp1* I/*Hae* III and *Apek* I/*Hae* III. The size-related traits from flowers were negatively related to the color-related traits. The 1191 SNPs from *Hinp1* I/ *Hae* III and 23 simple sequence repeats were used to establish a high-density genetic map of 19 homolog groups for *P. equestris*. In addition, 10 quantitative trait loci were highly associated with four color-related traits on chromosomes 2, 5 and 9. According to the sequence within the linkage disequilibrium regions, 35 candidate genes were identified and related to anthocyanin biosynthesis. In conclusion, we performed marker-assisted gene identification of aesthetic traits with GWAS in *Phalaenopsis* orchids.

## Introduction

The Orchidaceae is one of the largest families in angiosperm, with 27,315 species. *Phalaenopsis* is the most popular orchid genus, with approximately 92 species as well as 35,129 hybrids recorded for the registration in the Royal Horticultural Society^1^. Both *P. equestris* and *P. aphrodite* are two model orchid plants used in academic studies and are major breeding parents in orchid nurseries. The whole-genome sequences of *P. equestris*^2^ and *P. aphrodite*^3^ have been published and are available in Orchidstra^3^ (http://orchidstra.abrc.sinica.edu.tw) and OrchidBase^4^ (http://orchidbase.itps.ncku.edu.tw) , respectively. The genetic information for *P. equestris* has been used in several functional genomics studies of flower morphogenesis, pigmentation patterning, floral fragrances, stress response, etc.^5–12^. However, marker-assisted gene isolation has rarely been used for functional characterization of agricultural traits for *Phalaenopsis* breeding.

Marker-assisted selection (MAS) involves using molecular markers to support desired phenotypic selections in crop development. Next-generation sequencing technologies aim to effectively identify single nucleotide polymorphism (SNP) markers from ultra-throughput sequences. The method has revolutionized plant genotyping in crops and plant breeding^13,14^. To broaden next-generation sequencing use to large–genome crops, genotyping-by-sequencing (GBS) has been established and used to sequence pooled samples that identify the molecular markers and for the genotyping^15^. So far, GBS has been effectively used in genome-wide association study (GWAS) because of its cost-effectiveness and as an ultimate MAS^16^. Other applications of GBS are equally important in plants, such as study of genetic diversity, genetic linkage investigation, molecular marker detection, and genomic selection in breeding programs^14^. By genotyping large-size populations, GBS is an outstanding method for plant breeding, even without the information on reference genome sequences; examples of plants investigated are rapeseed^17^, lettuce^18^, switchgrass^13^, soybean^19^, and maize^20^.

GWAS is a useful and powerful approach for identifying genetic variations that underlie many important and complex phenotypes, especially quantitative trait loci (QTL) controlled by multiple genes^21^. In cassava, a useful GBS pipeline has been established to discover SNPs both within and among the mapping population and varied African cassava varieties, which improved the MAS programs to increase the disease resistance ability and the nutrition concerns^22^. Recently, several studies focused on GWAS for aesthetic floral traits even though it is not a major field for most crops. These studies involved rose^23^, cultivated sunflower^24^, woody plant *Prunus mume*^25^, and chrysanthemum^26^ and identified SNP markers associated with flower color and floral shape.

For *Phalaenopsis* breeding, both *P. equestris* and *P. aphrodite* are popular parents; in total, 35,129 hybrids have been registered by the Royal Horticultural Society^1^. *P. equestris* has small flowers 2.5 ~ 3.8 cm in floral diameter and various flower colors, including red flowers with red lip, red flowers with orange lip, white flowers with white lip, white flowers with yellow lip, and light blue-purple flowers. *P. aphrodite* has white and medium-size flowers 6 cm in diameter; it is the major breeding parent to offer pure-white flowers. The cross between *P. aphrodite* and *P. equestris* resulted in the F1 progeny *P.* Intermedia, containing red, pink, to white flowers with small to medium sizes. Therefore, *P.* Intermedia provides a good population for investigating floral aesthetic traits with MAS and GWAS. In addition, high-quality and well-annotated genome sequences have been published for both parent materials^2,3^ and could be used as reference genomes for SNP calling. However, GWAS has not been well established in *Phalaenopsis* orchids.

In this study, by using the valuable *P.* Intermediate population, we first constructed a genetic map of *P. equestris* by using SNPs obtained from GBS and previously identified simple sequence repeat (SSR) markers. We revealed the relationships by combining the SNPs and floral aesthetic traits to identify QTL that contribute to 4 different color-related traits in *Phalaenopsis*. This is the first report of the genetic map and flower color-related QTL in *Phalaenopsis* orchids.

## Results

### Distribution of various agricultural traits

**Figure 1:**
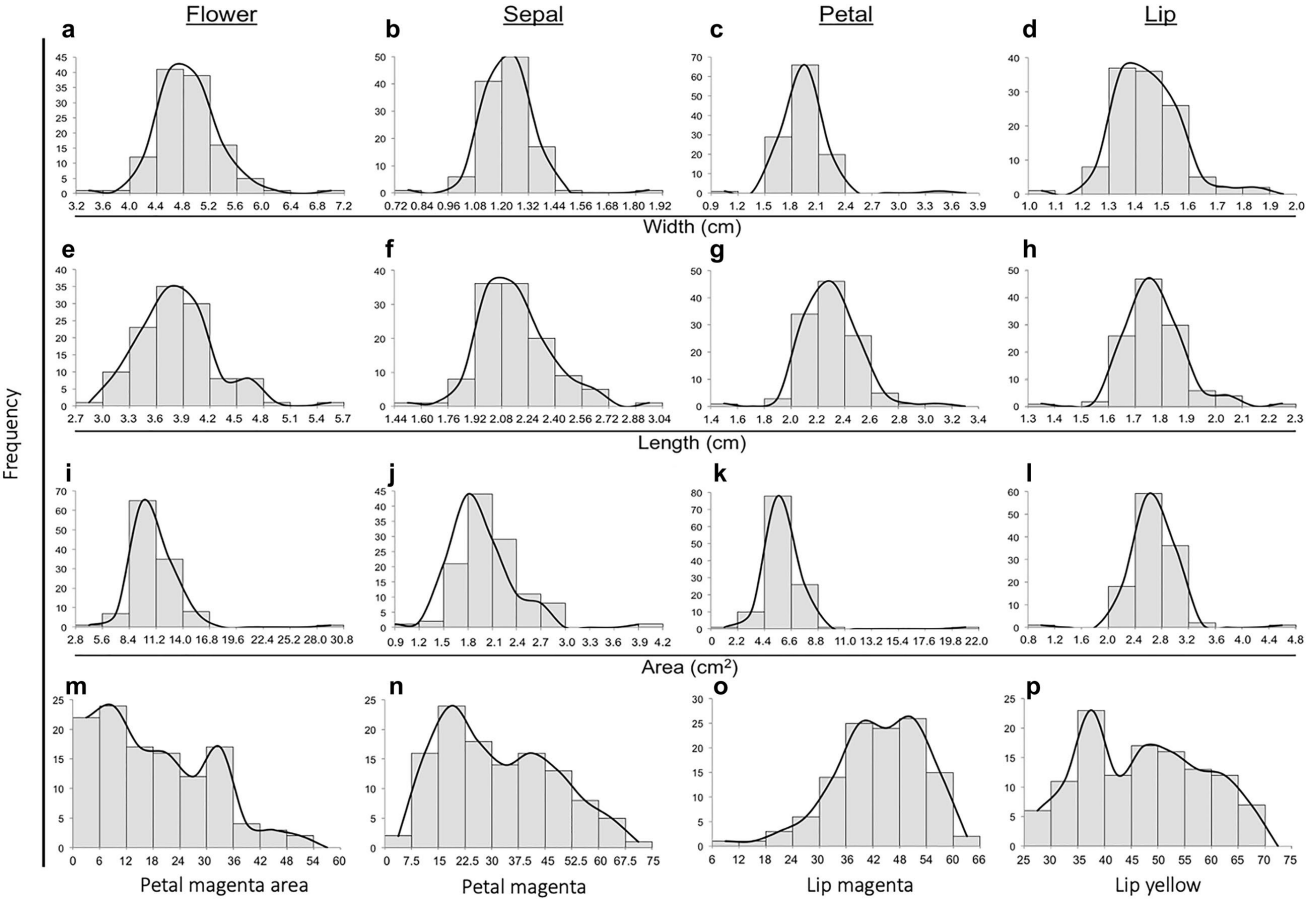
Distribution of flower aesthetic traits in *P.* Intermedia (**a**–**l**). For flower: a, e, I, m; for sepal: b, f, j, n; for petal: c, g, k, o; for lip: d, h, l, p. The aesthetic traits of width (**a**–**d**), length (**e**–**h**), and area (**i**–**l**). Frequency distribution of flower color-related traits in *P.* Intermedia (**m**–**p**). The color-related traits are magenta area in petal (**m**), magenta color in petal (**n**), red color in lip (**o**), and yellow color in lip (**p**).

**Figure 2:**
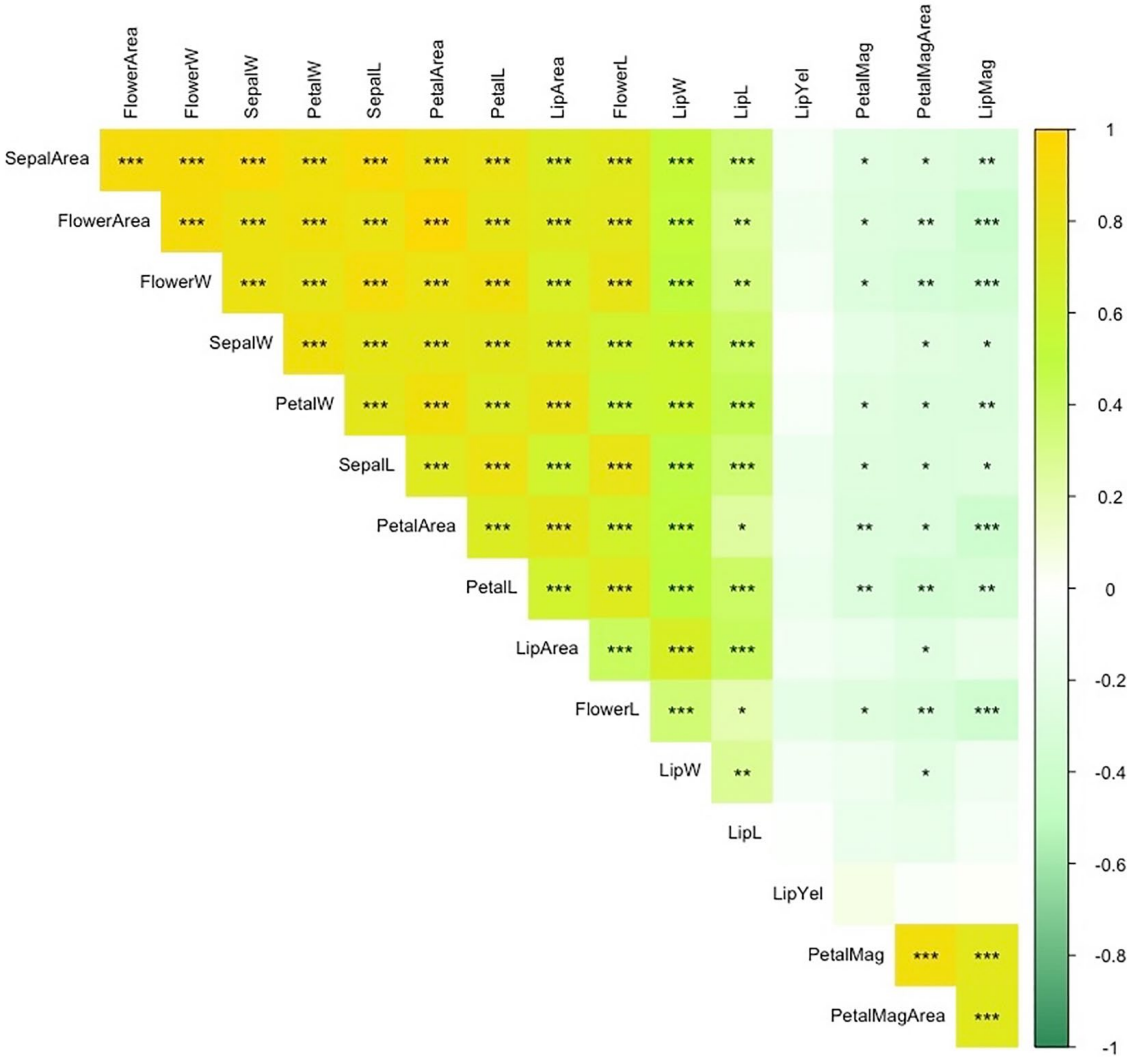
Correlation coefficients for all floral aesthetic traits. The yellow and green colors represent the positive and negative correlation coefficients estimated by Pearson correlation. *,**,***P < 0.05, 0.01, 0.001, respectively.

We recorded flower size and flower color-related phenotypes from the 117 F1 progenies of *P.* Intermedia. A total of 16 phenotypes were assessed, including 12 traits involved in flower size and 4 involved in color-related traits. The entire flower, sepal, petal and lip were measured for their width, length and area. The color-related traits included petal magenta area, petal magenta, lip magenta and lip yellow color. The flower size-related traits showed a normal distribution (Fig. 1a–l), but the flower color-related traits showed a complex distribution (Fig. 1–p). We assessed the relationship among all characteristics, and most of the flower size-related traits were positively correlated, with strong significant values, the strongest correlation (correlation coefficient = 0.96, p = 9.8584E−52) being between flower area and petal area (Supplementary Table S1, combination 1). In addition, most color-related traits were positively correlated with each other, with the highest correlation coefficient 0.89 (p = 5.6991E−32) between petal magenta and petal magenta area (Supplementary Table S1, combination 8). However, a low positive to negative correlation was found between size-related and color-related traits (Fig. 2; Supplementary Table S1).

### Genetic map and distribution of SNPs in the Phalaenopsis genome

**Figure 3:**
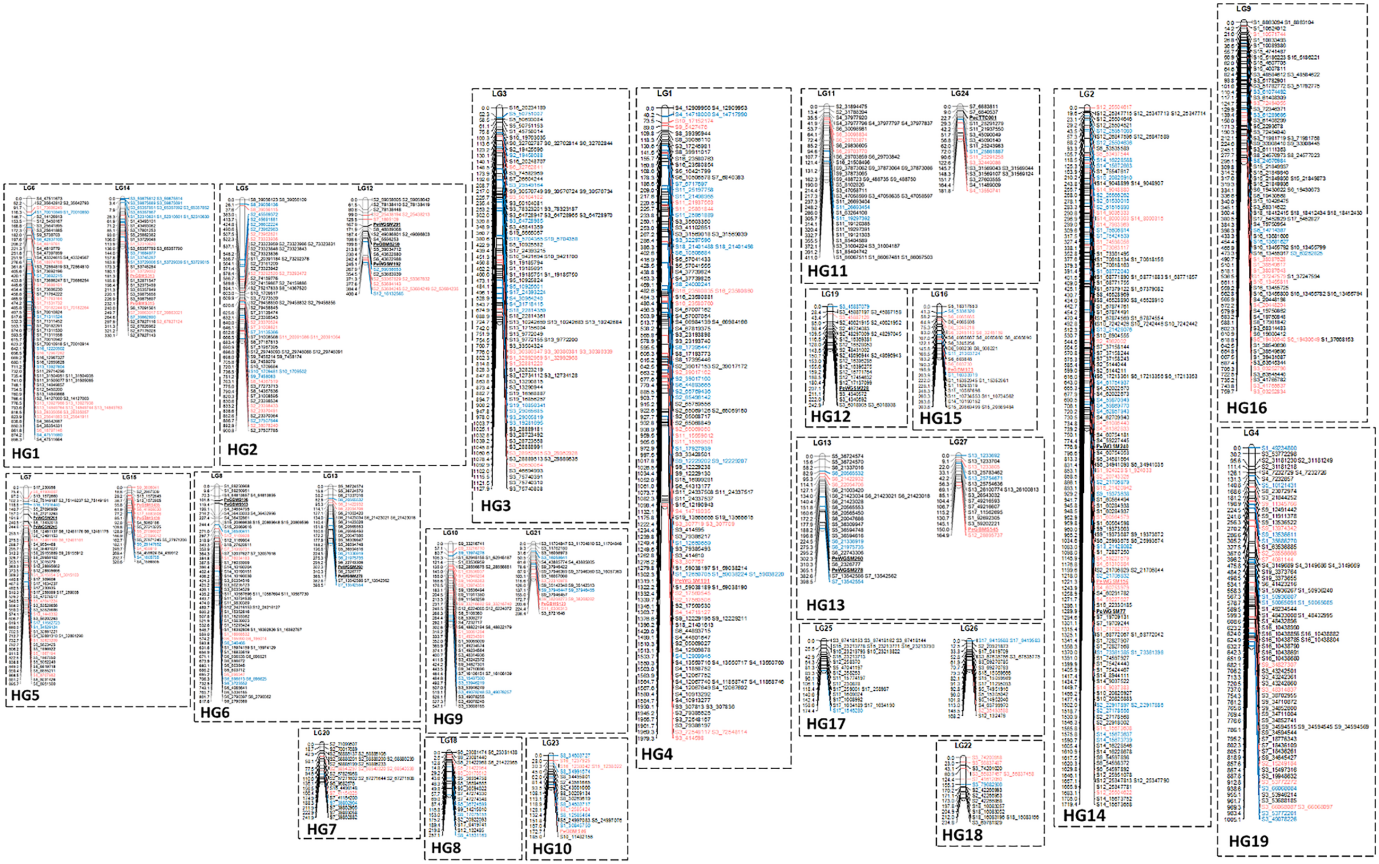
The genetic linkage map for *Phalaenopsis* that contains 19 potential homologous groups (HGs). The 19 HGs were assembled from 27 linkage groups (LGs) based on *P. equestris* BAC-end sequences (BESs).

With GBS, the SNP numbers obtained by using different restriction enzyme combinations of *Hinp1* I/Hae III and *ApeK* I/Hae III were 1,633 and 111,884 by Mi-seq and Hi-seq, respectively. A total of 1,191 SNPs from *Hinp1* I along with 23 SSRs previously identified by Dr. Wen-Luan Wu’s lab^27^ were successfully used to construct a genetic map for the F1 population of *P.* Intermedia (Supplementary Table S2), which revealed 27 linkage groups (LGs) (Supplementary Table S3). With the assistance of 21,350 BAC-end sequences (BESs)^27^, the 27 LGs were further assembled into 19 homologous groups (HGs) (Supplementary Table S4; Fig. 3). The number of total markers in each HG ranged from 15 in HG 19 to 144 in HG 14. These markers spread to a total map length of 15,192.05 cM, with a physical distance of 875,501 bp and average distance between two SNPs of 721 kb (Supplementary Table S4).

A total of 113,517 SNPs from the two enzyme digestions were used in GWAS. The SNP number in each chromosome ranged from 24,121 for chromosome 1 to 1636 for chromosome 19, and the average distance between SNPs ranged from 16,266 bp in chromosome 2 to 2849 bp in chromosome 19. The average distance between SNPs in the whole genome was 8854 bp (Table 1). To identify the genomic structure in the F1 population, we used multidimensional scaling (MDS) analysis based on the total SNPs above and showed that the genomic structure should be assessed in the following GWAS analysis (Supplementary Fig. S1).

**Table 1:** Distribution and frequency of single nucleotide polymorphisms (SNPs) identified using the genotyping-by-sequencing.

Chromosome	Chromosome length (bp)	Number of SNP in chromosome	Average distance between SNPs (bp)
1	258,396,435	24,121	10,713
2	224,508,944	13,802	16,266
3	70,575,877	8805	8015
4	67,241,588	6726	9997
5	49,315,034	8784	5614
6	39,338,091	7383	5328
7	36,994,609	5184	7136
8	35,905,232	4584	7833
9	30,537,433	3428	8908
10	29,494,005	3861	7639
11	25,713,836	2204	11,667
12	26,363,526	3707	7112
13	25,495,552	2286	11,153
14	22,079,116	2000	11,040
15	20,647,704	3648	5660
16	16,685,943	5094	3276
17	11,213,544	3806	2946
18	9,947,944	2458	4047
19	4,661,377	1636	2849
Total	1,005,115,790	113,517	8854

### GWAS results for flower color-related traits and candidate genes

**Figure 4:**
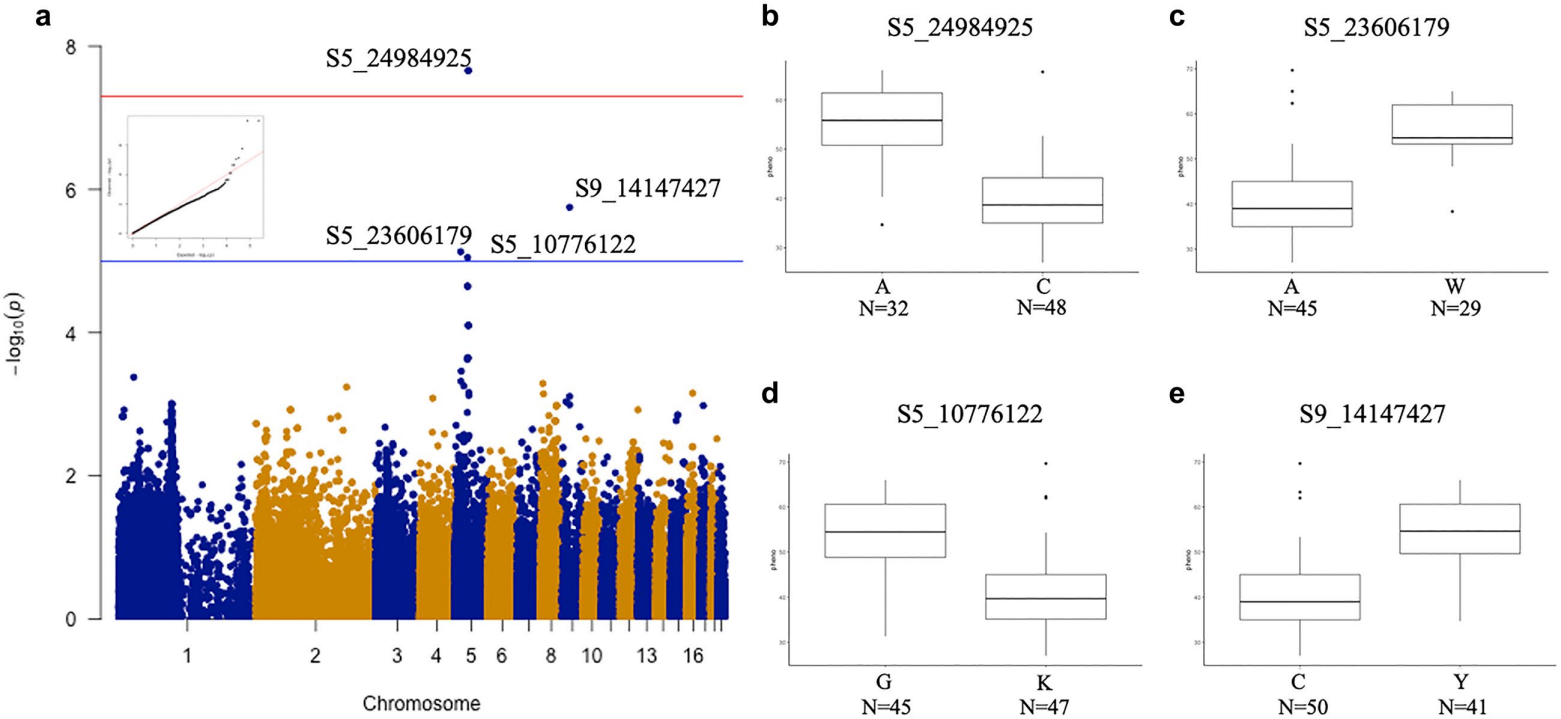
Genome-wide association study (GWAS) of lip yellow color. (**a**) Manhattan plot of lip yellow color, (**b**) difference in lip yellow color level by single nucleotide polymorphism (SNP) type at position S5-24984925, (**c**) SNP type at position S5-23606179, (**d**) SNP type at position S5-10776122, (**e**) SNP type at position S9-14147427. N indicates the individual number of this SNPs.

**Figure 5:**
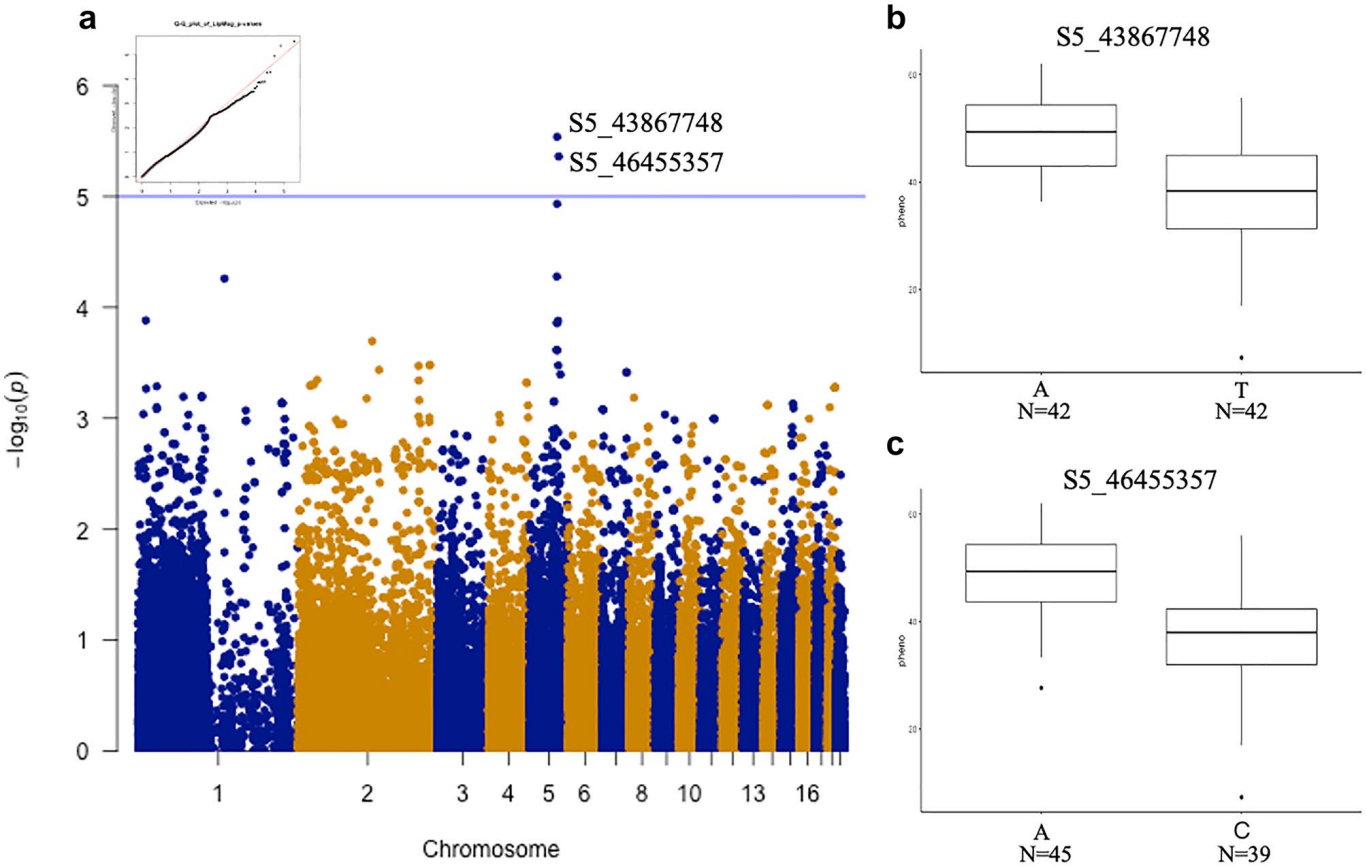
GWAS of lip magenta color. (**a**) Manhattan plot of lip magenta color, (**b**) difference in lip Magenta color level by SNP type at position S5-43867748, (**c**) SNP type at position S5-46455357. N indicates the individual number of this SNPs.

**Figure 6:**
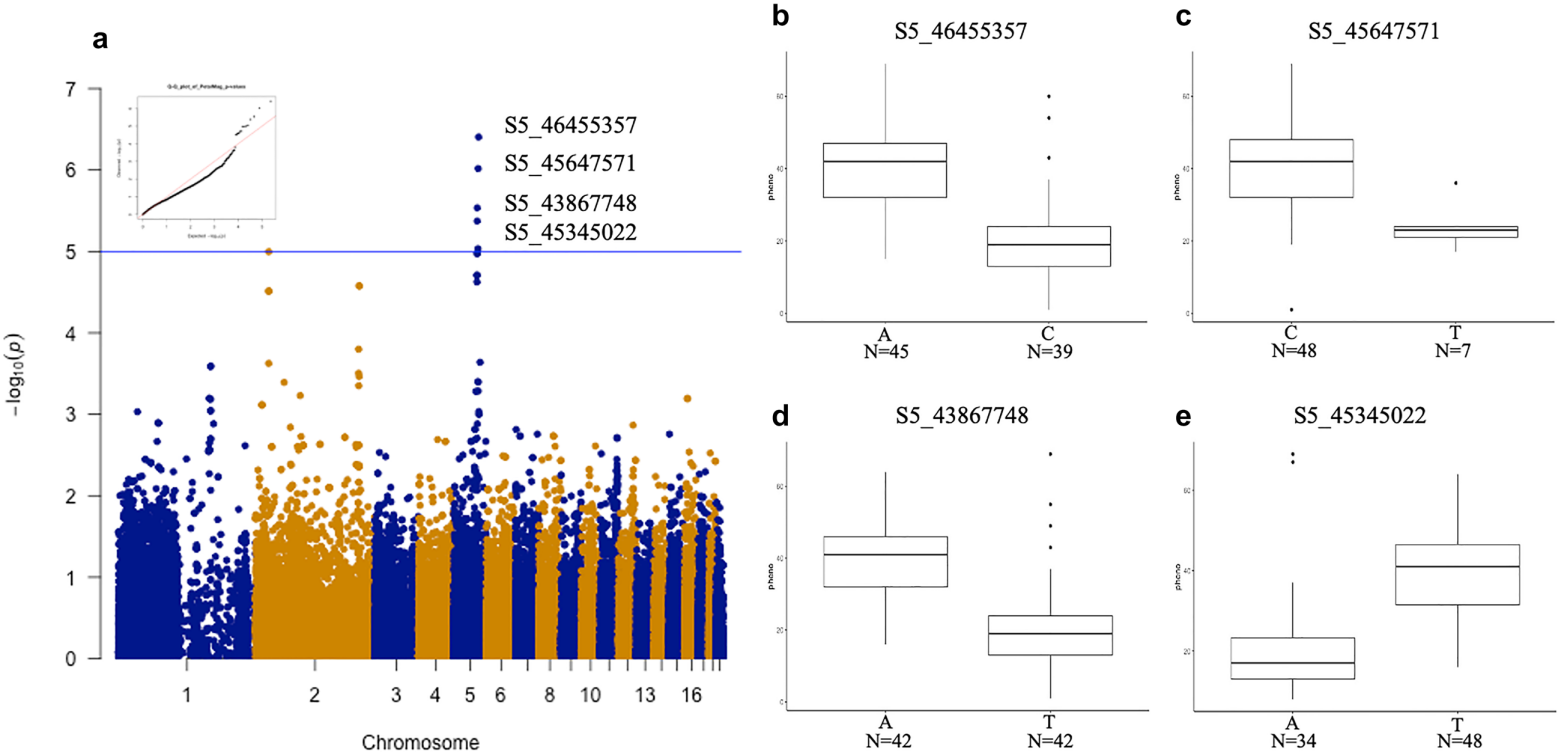
GWAS of petal magenta color. (**a**) Manhattan plot of petal magenta color, (**b**) difference in petal magenta color level by SNP type at position S5-46455357, (**c**) SNP type at position S5-45647571, (**d**) SNP type at position S5-43867748, (**e**) SNP type at position S5-45345022. N indicates the individual number of this SNPs.

**Figure 7:**
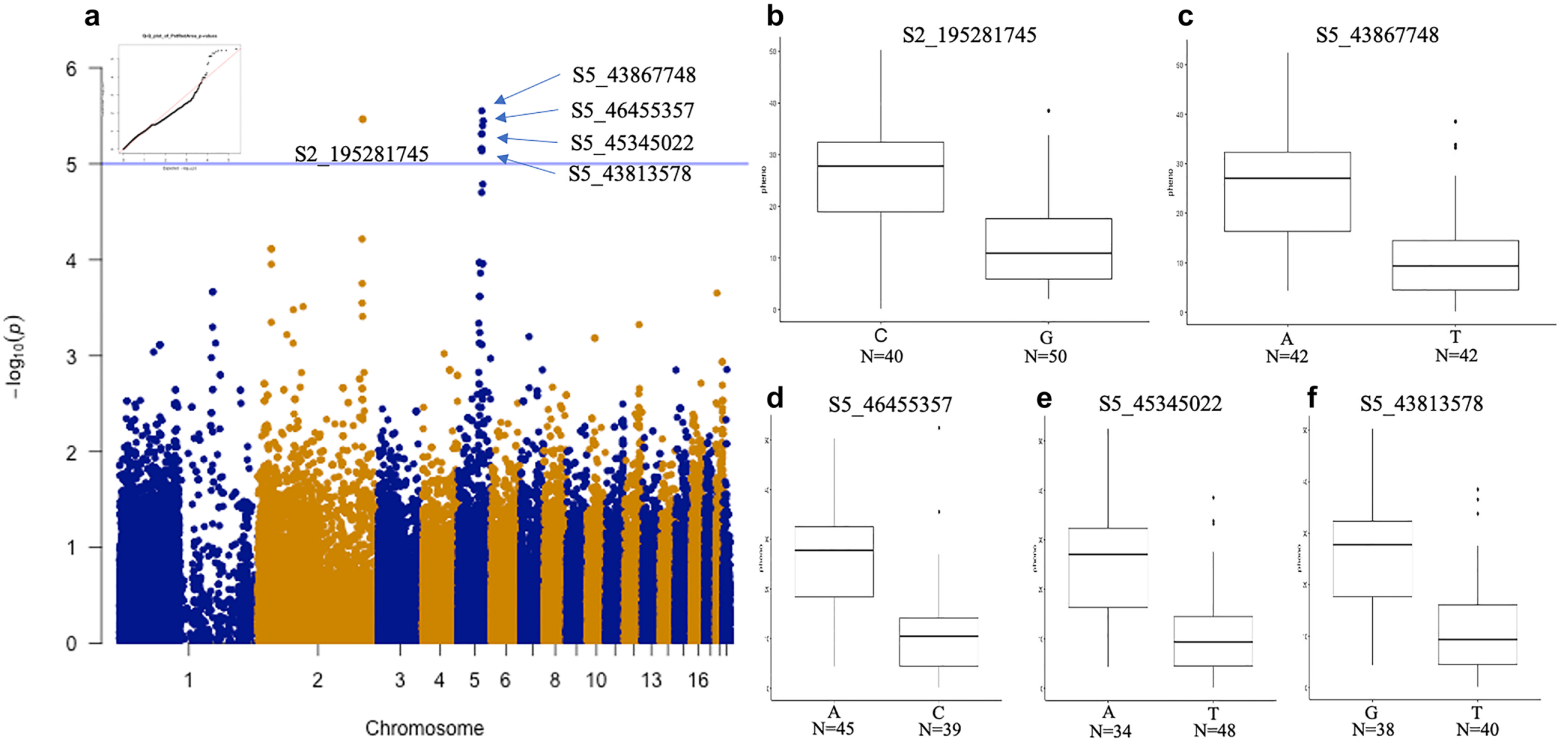
GWAS of petal magenta area. (**a**) Manhattan plot of petal magenta area, (**b**) difference in petal magenta area by SNP type at position S2-195281745, (**c**) SNP type at position S5-43867748, (**d**) SNP type at position S5-46455357, (**e**) SNP type at position S5-45345022, (**f**) SNP type at position S5-43813578. N indicates the individual number of this SNP.

GWAS was used to identify the SNPs correlated with floral aesthetic traits. Ten SNPs were identified for color-related traits contributing to phenotype variations for lip yellow color (LipYel), lip magenta color (LipMag), petal magenta color (PetalMag) and petal magenta area (PetalMagArea) (Figs. 4, 5, 6, 7). Of note, seven SNPs were associated with only one color-related trait: S2_195281745 on chromosome 2 and S5_43813578 on chromosome 5 were associated with the PetalMagArea trait, and SNP S5-10776122, S5-23606179, and S5-24982925 on chromosome 5 and S9-14147427 on chromosome 9 were associated with the LipYel trait. In addition, SNP S5_45647571 on chromosome 5 was related to the PetalMag trait. The remaining three SNPs affected multiple traits. For example, SNP S5_45345022 located on chromosome 5 could explain the variation in PetalMagArea and PetalMag traits. The SNPs S5_43867748 and S5_46455357 on chromosome 5 contributed to the traits LipMag, PetalMagArea and PetalMag.

We calculated the linkage disequilibrium (LD) for this population based on SNPs. The LD decay was 50 Kb, with r^2^ ˂ 0.12 (Supplementary Fig. S2 across all chromosomes. The sequence within 50 Kb upstream and downstream of the significant SNPs was then extracted and screened for candidate genes by using a BLAST search against the NCBI nr database (https://blast.ncbi.nlm.nih.gov/Blast.cgi). Matched genes with total score > 150 were kept and narrowed down to 35 candidate genes based on the BLAST targeted species of *P. equestris*. Anthocyanin biosynthesis-related genes, such as MYB genes and flavanone 3-hydroxylase (F3H5), were identified within the LD region of the associated SNPs S2_195281745, S5_10776122, S5_43813578 and S5_45647571. Other genes including a MADS box protein (*SUPPRESSOR OF OVEREXPRESSION OF CO 1, SOC1*), ABC transporter B family, sugar transport protein, auxin-binding protein, tubby-like F-box protein and GEM-like protein were also identified within the LD regions related to the phenotype traits LipYel, PetalMagArea, LipMag and PetalMag (Table 2).

**Table 2:** Candidate genes located in the upstream and downstream region of linkage disequilibrium to trait-associated SNPs based on the NCBI blast.

Associated SNP	Chr	Position	Associated Trait	r2	*p* value	Candidate gene	E value	Total Score
S5_10776122	5	10,776,122	LipYel	0.26	7.41E−06	*Phalaenopsis equestris* putative amidase C869.01 (LOC110029361), mRNA *Phalaenopsis equestris* flavanone 3-hydroxylase (F3H) gene, promoter region and 5' UTR	0 1.00E−50	3334 334
S5_23606179	5	23,606,179	LipYel	0.33	8.91E−06	*Phalaenopsis equestris* ammonium transporter 1 member 2-like (LOC110038906), mRNA *Phalaenopsis equestris* MADS box protein (SOC1) gene, complete cds	0 0	4556 2726
S5_24984925	5	24,984,925	LipYel	0.51	2.19E−08	*Phalaenopsis equestris* sulfoquinovosyl transferase SQD2-like (LOC110027696), transcript variant X2, mRNA *Phalaenopsis equestris* phosphoenolpyruvate carboxykinase (ATP) (LOC110027691), mRNA *Phalaenopsis equestris* ABC transporter B family member 26, chloroplastic (LOC110027673), mRNA	0 0 3.00E−57	1593 4899 1452
S9_14147427	9	1,414,742 7	LipYel	0.36	1.77E−06	*Phalaenopsis equestris* GEM-like protein 5 (LOC110032887), mRNA *Phalaenopsis equestris* cysteine-rich and transmembrane domain-containing protein A-like	0.00E+0 0 6E-114	2215 587
S2_195281745	2	195,281,745	PetalMagAre a	0.35	3.43E−06	*Phalaenopsis equestris* histone H2B.1-like (LOC110024163), mRNA *Phalaenopsis equestris* transcription factor MYB52-like (LOC110024256), mRNA *Phalaenopsis equestris* agamous-like MADS-box protein AGL65 (LOC110024229), transcript variant X6, mRNA *Phalaenopsis equestris* MADS box protein (SOC1) gene, complete cds	0 0 0 4E−175	1031 2298 2879 2416
S5_43813578	5	43,813,578	PetalMagAre a	0.38	4.88E−06	*Phalaenopsis equestris* subtilisin-like protease SBT1.4 (LOC110018389), mRNA *Phalaenopsis equestris* keratin-associated protein 4–6-like (LOC110018400), mRNA *Phalaenopsis equestris* MADS box protein (SOC1) gene, complete cds *Phalaenopsis equestris* uncharacterized LOC110025306 (LOC110025306), mRNA *Phalaenopsis equestris* sugar transport protein 8-like (LOC110025176), mRNA *Phalaenopsis equestris* MYB11 promoter region *Phalaenopsis equestris* retrotransposon HORT1 hypothetical protein gene, complete cds	0 0 0 0 6E−34 2.00E−13 2.00E−13	5451 2572 4336 802 165 194 194
S5_45647571	5	45,647,571	PetalMag	0.41	9.59E−07	*Phalaenopsis equestris* 40S ribosomal protein S3-3-like (LOC110023171), mRNA *Phalaenopsis equestris* eukaryotic peptide chain release factor GTP-binding subunit ERF3A (LOC110023165), mRNA *Phalaenopsis equestris* MADS box transcription factor 5 (MADS5) gene, promoter region and 5' UTR *Phalaenopsis equestris* flavanone 3-hydroxylase (F3H) gene, promoter region and 5' UTR	0 5E−139 3.00E−42 1.00E−30	2132 3574 193 154
S5_45345022	5	45,345,022	PetalMagAre a PetalMag	0.39 0.37	4.00E−06 9.26E−06	*Phalaenopsis equestris* uncharacterized protein At4g06744-like (LOC110023166), mRNA *Phalaenopsis equestris* auxin-binding protein ABP19a-like (LOC110023170), mRNA *Phalaenopsis equestris* MADS box transcription factor 4 (MADS4) gene, promoter region and complete cds	0 0 2E-124	3827 3510 466
S5_43867748	5	43,867,748	LipMag PetalMagAre PetalMag	0.31 0.32 0.35	2.89E−06 2.80E−06 2.91E−06	*Phalaenopsis equestris* cytochrome P450 71A1-like (LOC110018390), mRNA *Phalaenopsis equestris* serine/threonine-protein kinase RIPK-like (LOC110018387), mRNA *Phalaenopsis equestris* MADS box protein (SOC1) gene, complete cds *Phalaenopsis equestris* keratin-associated protein 4–6-like (LOC110018400), mRNA *Phalaenopsis equestris* probable serine/threonine-protein kinase PBL23 (LOC110037276), mRNA	0 0 0 0 1E−164	3333 2651 4336 1119 599
S5_46455357	5	46,455,357	LipMag PetalMagAre PetalMag	0.30 0.31 0.38	4.36E−06 3.57E−06 3.93E−07	*Phalaenopsis equestris* digalactosyldiacylglycerol synthase 1, chloroplastic (LOC110019456), mRNA *Phalaenopsis equestris* monoacylglycerol lipase-like (LOC110019459), mRNA *Phalaenopsis equestris* tubby-like F-box protein 1 (LOC110029707), mRNA	0.00E+00 0.00E+00 4.00E−31	5407 3513 156

## Discussion

### Flower size-related traits are highly related

Flower size and color are important aesthetic traits for ornamental flowers. We estimated the correlation of each group of flower traits, such as size-related traits and color-related traits, and found significant positive correlations within each group. This finding make sense that the width and length of each flower organ affect the entire flower size. Previous study showed flower disc diameter positively correlated with disc area in sunflower^24^. However, we found significant negative correlations between size-related and color-related traits. Similarly, a negative relation was discovered between petal length and red accumulation in *Camissoniopsis cheiranthifolia*^28^. In addition, carotenoid content in cultivated sunflower was found negatively correlated with flower size traits^24^. These results suggest that the larger the size, the lighter the yellow/red color for the F1 population of *P.* Intermedia. However, more studies are needed to determine the control mechanism between flower size and color.

### Genetic map for P. equestris

Several genetic maps are available for Orchidaceae, with different molecular markers, including *Dendrobium*, *Vallina,* and *Phalaenopsis*. In *Dendrobium*, 349 polymorphic loci are identified from the cross between *Dendrobium officinale* × *D. aduncum* with a total length of 1580.4 cM and 19 LGs, covering 71% of the genome^29^. In addition, specific locus-amplified fragment sequencing was used recently to build genetic maps: a genetic map with high density was developed by the cross of *D. moniliforme* and *D. officinale*, with a longer length map of 2737.49 cM and 19 LGs^30^. In vanilla, a genetic linkage map with total length of 1035.85 cM and 18 LGs was built based on 225 amplified fragment length polymorphism markers^31^. In *Phalaenopsis*, 2905 SNP markers from restriction site-associated DNA sequencing with the cross between *P. aphrodite* and *P. modesta* was used to build a genetic map with a total length of 3075.8 cM and 22 LGs, with 85% coverage of the *P. aphrodite* genome^3^. In this study, we used 1191 SNPs and 23 SSRs to successfully construct a genetic map of 19 HGs with length of 15,192.05 cM and 875,501 kb, which is equivalent to the chromosome number of *P. equestris,* that covered 75.5% of the genome region. With the assistance of abundant BESs, the linkage map was assembled into 19 homologous maps.

### Flower color-related QTL

GWAS is a state-of-art study for identifying genomic loci associated with desired traits^32^. Understanding the numbers and locations of loci regulating a trait is important to resolve genetic architecture and is valuable to plan a successful breeding strategy. Therefore, combining a high-throughput genotyping technology and a precise phenotyping method provides precise results for the GWAS analysis. The GBS approach we used provides rapid, cheap, high-throughput, and reliable results for genotyping hundreds of individuals in one population^33^. We obtained 113,517 SNPs after combining two different GBS libraries (*Hinp1* I/*Hae* III and *ApeK* 1/*Hae* III), with an average distance between 2 markers of 8854 bp/SNP, and these markers supported a great resolution for the GWAS. However, no SNPs were identified for the association with size-related traits. Increasing the population size with enlarged flower size variations may be needed for future GWAS.

### Candidate genes of red color-related QTL

Ten SNPs showed significant associations with the 4 color-related traits, and 3 were associated with more than one trait (Table 2). Pleiotropy has been found a common phenomenon in floral trait QTL^34^. QTL with pleiotropy may contribute to similar phenotypes, including petal length, sepal length and stamen length, or affect the phenotype for different traits, such as flower length, scent and pigmentation^34^. In our study, the situation of pleiotropy showed that the SNPs S5_45345022, S5_43867748 and S5_46455357 contributed to PetalMagArea and PetalMag, affecting the color magenta and the color distribution by the same QTL.

The regulation of flower color is a complex network; many factors affect the performance. For instance, plant hormones^35^, sugar transportation^36^, environmental stress^37^, retrotransposon activation^38^, carotenoid and anthocyanidin biosynthesis pathway^39^, and regulatory genes such as the MYB family^40^ and MADS-box family^41^ are all involved in the flower color regulation network. We identified 35 candidates within 10 QTL for 4 different color-related traits after a BLAST search of the NCBI nr database; 26 candidate genes located within 6 QTL related to 3 different traits: PetalMagArea, PetalMag and LipMag (Table 2). These genes include *MYB52*-like gene and the *MYB11* promoter region around the significant SNPs S2_195281745 and S5_43813578, respectively, correlated with the PetalMagArea trait. Different genes in the MYB family have the function of determining red color in floral tissue by regulating anthocyanin biosynthesis; examples are *OgMYB1* in *Oncidium*^42^, *GmMYB-G20-I* in soybean^43^, and the *MYB117* promoter in the hybrid poplar *Populus tremula* × *tremuloides*^44^. *MYB108* regulates the color of petal, stigma, calyx and bud in the woody plant *Prunus mume*^25^*,* and R2R3*-MYB* from *Phalaenopsis* controls floral pigmentation patterning^11^. In addition, MADS-box genes are involved in the anthocyanin pathway in potato^41,45^ and *Phalaenopsis* orchid^46^. We identified the MADS-box family genes *AGL65*, *MADS5*, and *MADS4* within the QTL of the SNPs S2_195281745, S5_45647571, and S5_45345022, respectively (Table 2), so they may not only regulate floral morphogenesis but also be involved in the anthocyanin biosynthesis pathway.

Sucrose activation^36^ and sucrose signaling pathways^47^ also affect anthocyanin accumulation. We identified a sugar transport protein 8-like gene within the LD region of the significant SNP S5_43813578. In addition, auxin, the important plant hormone for plant growth and development, has a role in anthocyanin modification in apple^48,49^. We found an auxin-binding protein *ABP19a*-like gene within the QTL S5_45345022 for PetalMag and PetalMagArea traits. Cytochrome P450 is required for fully activating the formation of flower color^50,51^. We identified a cytochrome P450 71A1-like gene associated with the SNP S5_43867748 for LipMag, PetalMag and PetalMagArea traits. In addition, 2 serine/threonine-protein kinases, RIPK-like and PBL23, were identified within the LD region associated with the SNP S5_43867748 for LipMag, PetalMag and PetalMagArea traits. This result is consistent with a previous study of rose, in which a serine/threonine-protein kinase, PBS1, was a candidate gene modifying anthocyanin content in petal^23^. Insertion of an *HORT1* retrotransposon controlling flower color was confirmed recently^38^. In our study, a short fragment of *HORT1* was found within the QTL S5_45647571.

### Candidate genes of yellow color-related QTL

Nine candidate genes located within 4 QTL contributing to the LipYel trait included flavanone 3-hydroxlase (*F3H*) gene and sulfoquinovosyl transferase *SQD*-2 like gene (Table 2). A short fragment of *F3H* was found in the QTL S5_45647571, also associated with the PetalMag trait. Thus, *F3H* may be involved in regulating both red and yellow colors in *Phalaenopsis* flower. The role of *F3H* in the anthocyanin biosynthesis pathway has been investigated carefully, and it was found to have an important role regulating red color accumulation in flower^37,39,52^. For regulating yellow color, less expression of *F3H* was found in yellow fruit of *Fragaria vesca* as compared with red fruit^53^, and minor amino acid differences caused no anthocyanin accumulation in *Gentiana lutea*^54^. In addition, *F3H* loss by antisense suppression produced a yellow carnation^55^. The evidence from these previous studies indicates that *F3H* may be involved in red and yellow flower color regulation and support our findings in this study. These candidate genes within the QTL most likely participate in and support their roles for these color-related traits.

Overall, most candidate genes identified by GWAS were correlated with anthocyanin biosynthesis, and the rest of them were discovered for the first time to be involved in regulating flower color; examples are ammonium transporter 1, the ABC transporter B family, GEM-like protein, histone *H2B.1*-like, and digalactosyldiacylglycerol synthase 1. *SOC1*, a MADS-box gene, was identified within QTL associated with all color-related traits, with a high total score and significant E-value, so *SOC1* may participate in the flower color regulation pathway. *SOC1* functions to regulate flowering time and floral development^56,57^. Our report is the first to reveal its function related to flower color regulation, possibly by regulating very upstream signals. However, more experiments are needed to confirm this.

In conclusion, this study provides the first marker-assisted gene identification of important agricultural traits in *Phalaenopsis* with GWAS and found that the associated SNPs could be used as selection markers for breeding programs of *Phalaenopsis* orchids.

## Materials and methods

### Plant materials and phenotyping

A total of 117 individuals from the F1 generation *P.* Intermedia and their parents, *P. aphrodite* and *P. equestris*, were used for both genotyping and phenotyping in this study. We analyzed the aesthetic phenotypes of these plants with the flower size-related traits, including width, length, and area of flower, sepal, petal, and lip and flower color-related traits and the red area in petal, petal red, lip red, and lip yellow. The traits for flower colors were collected with their CMYK values, magenta and yellow corresponding to red and yellow colors, respectively, in plant flower color. All plants were grown under normal light and the temperature was controlled between 23 °C and 27 °C in the greenhouse at Taiwan Sugar Corp. (Tainan, Taiwan). The correlation among all traits were estimated by using Pearson correlation in R^58^. The parental materials *P. aphrodite* and *P. equestris*, and the *P.* Intermedia, offspring derived from the cross between them, are all commercially available. The authenticity of these materials was verified by Taiwan Sugar Corp. These materials have been deposited in the greenhouse at National Cheng Kung University. Experimental research and field studies of plants, including the collection of plant material, comply with relevant institutional, national, and international guidelines and legislation.

### Two- enzyme GBS approach

DNA extraction involved using the Qiagen DNeasy 96 Plant kit (Cat. no. 69181, Qiagen) following the manufacturer’s protocol. For the two-enzyme GBS approach, *HinpI 1* and *Ape*K1 were paired with *HaeIII* for library preparation following a previous protocol^59^ with minor modifications. Single-end sequencing of multiplex GBS libraries involved using Mi-seq (for *HinpI* 1 library) and Illumina HiSeq2000 (for *ApeK* I library) platforms. Illumina sequence read processing, mapping, and SNP calling involved using the TASSEL-GBS pipeline^60^. The raw data of the above libraries and SNPs were uploaded into NCBI (accession no. GSE176215).

### Genetic map construction

To construct the basic genetic map, 23 SSRs that were polymorphic between the two parents and 1,191 SNPs from the *HinpI* 1 GBS library were collected. The OneMap^61^ package implemented in R^58^ was used to conduct recombination fractions and linkage phase estimation and construct a linkage map. The linkage groups (LGs) were identified by a 2-point test, then the genetic map was obtained. Any pairwise markers with logarithm of odds (LOD) score > 4.0 and recombination fraction < 0.50 were considered linked between any pairwise markers. The ordering algorithms was applied to each group. An exhaustive search was performed with the “compare” function for groups with < 6 markers to obtain the best order. The “order.seq” command was used for groups with > 6 markers. The best order was used as a structure for the following inclusion of new markers. Once these groups were obtained, the “try.seq” function was used to confirm markers linked over unlinked according to the initial procedure and that likely participated in the pre-ordered groups. The LGs with > 5 markers were reconstructed by using the “ripple” algorithm within a sliding window of 5 markers as the final step. Manual adjustment was used as required throughout the map-building process. In total, 27 LGs were sketched by using MAPCHART 2.30^62^. The constructed genetic maps then were rearranged according to SSRs and SNP markers that corresponded to 19 putative groups of scaffolds, which were grouped in parallel with the 21,350 BAC-end sequences (BESs) from pair-end sequencing. The homologous groups (HGs) were established from LGs that contained markers from the same group of scaffolds.

### GWAS

The following analyses involved using TASSEL5.0^60^. The SNP calling involved using Bowtie 2 in TASSEL5.0 with default settings. A kinship matrix and principal component analysis (PCA) was used with all SNPs. The mixed linear model (MLM) was used to identify the association between traits and SNPs:

$$ y = X\beta + Zu + e $$

where y indicates the phenotype observed and stored as a vector, β an unknown fixed effect shown as a vector as well, and genetic marker and population structure (Q) are counted here. “u” is a random additive genetic effect from multiple background QTL for individuals but is an unknown vector. X and Z are the known design matrices. “e” is an unobserved vector of random residual.

### LD estimation

For LD estimation, SNPs with the fewest missing data (NA) among all samples were selected every 5000 bp, then the LD was estimated among each of the collected SNPs. The LD plot and LD estimation involved using R^58^. The estimated LD between any two loci was based on comparing the observed and expected frequency of one haplotype, as follows:

$$ {\text{D}}_{{{\text{AB}}}} = {\text{P}}_{{{\text{AB}}}} - {\text{P}}_{{\text{A}}} {\text{P}}_{{\text{B}}} $$

where P_AB_ indicates the frequency of observed haplotype and P_A_P_B_ the frequency of the expected haplotype from two loci.

### Candidate gene identification

To predict the candidate genes around the significant associated SNPs, sequences of 50 Kb upstream and downstream around the most significant associated SNPs were extracted based on the LD decay region. The extracted sequences were uploaded to NCBI for BLAST analysis with default settings, and only genes with a total score > 150 and belonging to *P. equestris* were selected as candidates.

## Supplementary Information

Supplementary Information.

## Data Availability

The datasets generated in this study are available in NCBI with the accession number GSE176215.
