# Supplementary material for: High-density genetic map and genome-wide association studies of aesthetic traits in Phalaenopsis orchids
Source: Sci Rep. 2022 Feb 28;12:3346. doi: 10.1038/s41598-022-07318-w (PMC8885740; doi:10.1038/s41598-022-07318-w)
Supplement: Supplementary file 1 — Supplementary Information. [file 41598_2022_7318_MOESM1_ESM.pdf]

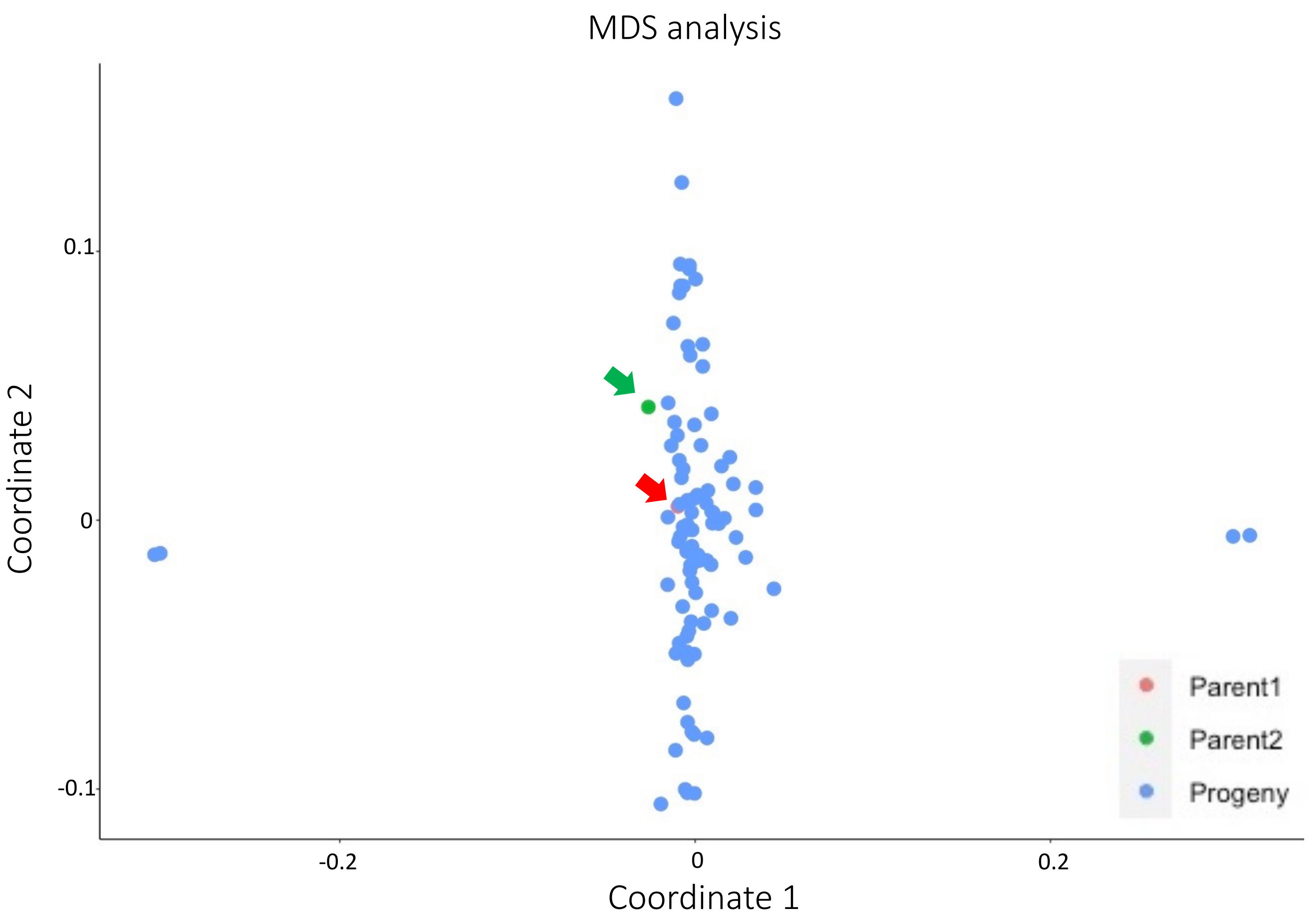

Supplementary Figure S1. Multidimensional scaling (MDS) analysis based on single nucleotide polymorphisms (SNPs) from two genotyping-by-sequencing (GSB) analyses. (Hsu et al. 2021)

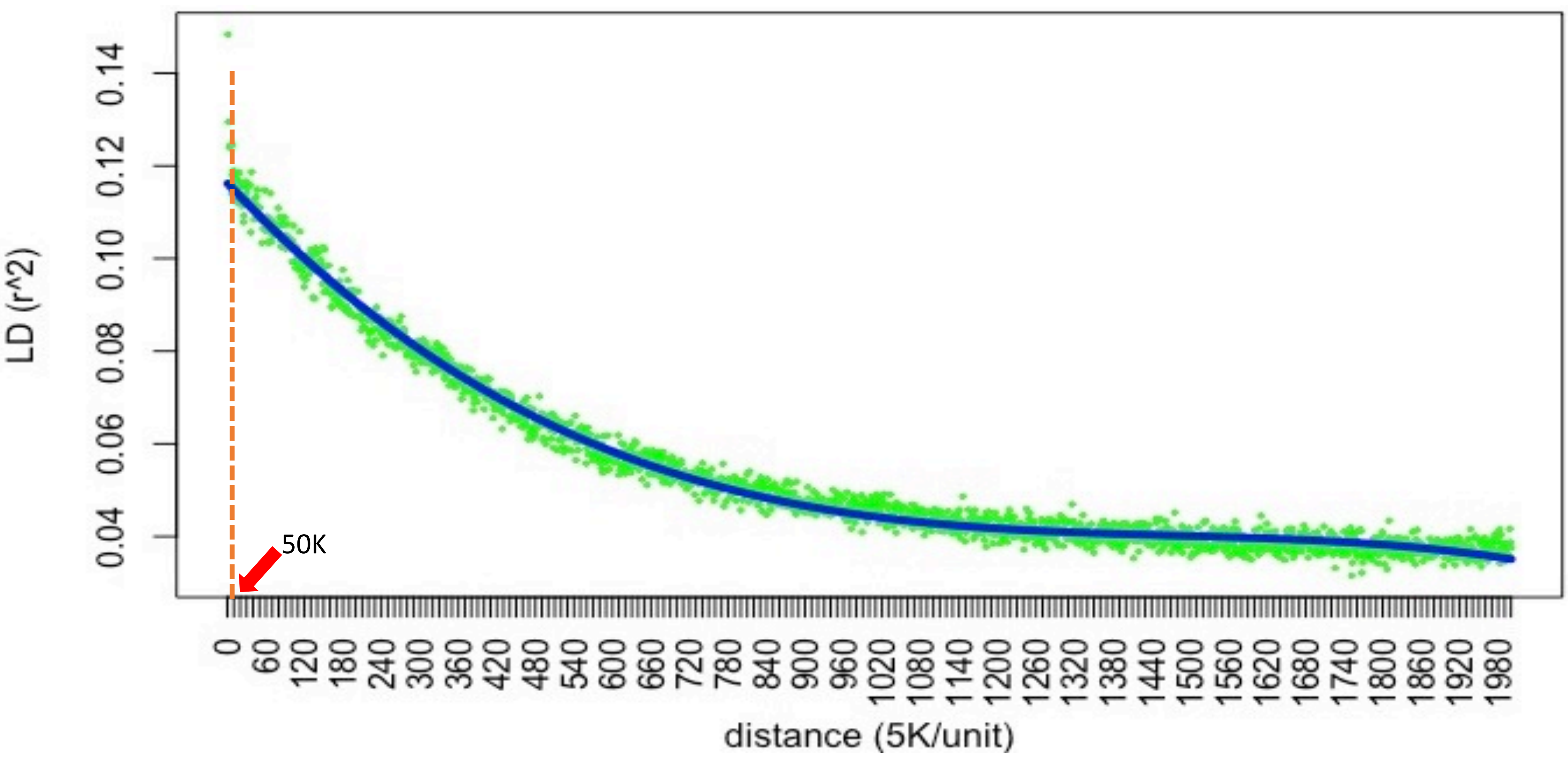

Supplementary Figure S2. Linkage disequilibrium decay plot for 117 individuals based on SNPs from two GBS analyses.(Hsu et al. 2021)

Supplementary Table S1. Correlation coefficient among all floral aesthetic traits.

Combination	Trait 1	Trait 2	Correlation coefficient	p-value
1	FlowerArea	PetalArea	0.96	9.8584E-52
2	SepalW	SepalArea	0.94	6.5605E-44
3	SepalL	SepalArea	0.94	4.2734E-43
4	FlowerW	SepalArea	0.93	1.7938E-38
5	FlowerArea	SepalArea	0.92	2.0824E-37
6	FlowerW	FlowerArea	0.92	5.2281E-37
7	FlowerW	SepalL	0.90	1.8889E-33
8	PetalMag	PetalMagArea	0.89	5.6991E-32
9	SepalArea	PetalW	0.88	1.0687E-29
10	PetalW	PetalArea	0.88	1.64E-29
11	FlowerW	PetalL	0.88	3.2791E-29
12	SepalW	PetalW	0.87	9.2184E-29
13	FlowerArea	PetalW	0.87	1.6116E-28
14	SepalArea	PetalArea	0.86	1.4851E-27
15	FlowerW	SepalW	0.85	1.7441E-26
16	FlowerArea	SepalW	0.85	2.5157E-26
17	SepalArea	PetalL	0.85	7.5067E-26
18	FlowerArea	SepalL	0.85	9.2969E-26
19	SepalL	PetalL	0.85	1.872E-25
20	FlowerW	PetalArea	0.84	5.5431E-25
21	FlowerL	SepalL	0.82	5.8328E-23
22	FlowerW	FlowerL	0.81	2.9466E-22
23	PetalW	LipArea	0.81	4.5791E-22
24	FlowerW	PetalW	0.81	6.9766E-22
25	SepalW	PetalArea	0.81	1.2476E-21
26	FlowerArea	PetalL	0.80	2.0406E-21
27	SepalW	SepalL	0.80	2.6157E-21
28	SepalW	PetalL	0.79	4.2783E-20
29	PetalArea	LipArea	0.78	1.4289E-19

30	SepalL	PetalW	0.78	1.8395E-19
31	FlowerL	FlowerArea	0.78	3.6158E-19
32	LipMag	PetalMag	0.77	1.1067E-18
33	FlowerL	SepalArea	0.77	1.6234E-18
34	LipMag	PetalMagArea	0.77	2.4223E-18
35	FlowerArea	LipArea	0.76	2.9053E-18
36	SepalL	PetalArea	0.75	2.06E-17
37	FlowerL	PetalL	0.75	5.1772E-17
38	PetalW	PetalL	0.74	7.1604E-17
39	SepalW	LipArea	0.74	1.3416E-16
40	SepalArea	LipArea	0.72	9.7395E-16
41	PetalL	PetalArea	0.71	6.6615E-15
42	FlowerW	LipArea	0.70	3.8254E-14
43	LipW	LipArea	0.69	8.299E-14
44	FlowerL	PetalArea	0.65	5.4504E-12
45	FlowerL	SepalW	0.64	1.8116E-11
46	PetalL	LipArea	0.63	2.8382E-11
47	SepalL	LipArea	0.63	3.9651E-11
48	PetalW	LipW	0.61	2.3886E-10
49	SepalW	LipW	0.60	4.4102E-10
50	FlowerL	PetalW	0.59	1.5119E-09
51	SepalArea	LipW	0.57	7.2914E-09
52	FlowerArea	LipW	0.55	3.1565E-08
53	PetalArea	LipW	0.52	1.9242E-07
54	FlowerW	LipW	0.51	2.4719E-07
55	PetalL	LipW	0.49	1.0063E-06
56	SepalL	LipW	0.48	1.5682E-06
57	PetalW	LipL	0.44	1.2913E-05
58	LipL	LipArea	0.42	3.4169E-05
59	FlowerL	LipArea	0.42	5.1203E-05
60	SepalW	LipL	0.40	8.5349E-05
61	PetalL	LipL	0.40	0.00010528

62	SepalL	LipL	0.37	0.00036354
63	SepalArea	LipL	0.37	0.00041685
64	FlowerL	LipW	0.36	0.00060408
65	FlowerW	LipL	0.33	0.00156091
66	FlowerArea	LipL	0.31	0.00329129
67	LipW	LipL	0.27	0.00987444
68	PetalArea	LipL	0.24	0.0223141
69	FlowerL	LipL	0.21	0.04991455
70	LipYel	PetalMag	0.07	0.51650471
71	LipMag	LipYel	0.01	0.90589087
72	SepalW	LipYel	-0.01	0.92255616
73	LipL	LipYel	-0.02	0.87972449
74	LipYel	PetalMagArea	-0.02	0.85000798
75	PetalW	LipYel	-0.06	0.58544188
76	LipL	LipMag	-0.06	0.55450118
77	SepalArea	LipYel	-0.08	0.46384119
78	FlowerW	LipYel	-0.08	0.4469432
79	LipW	LipYel	-0.09	0.38404017
80	LipArea	LipYel	-0.11	0.31635108
81	LipW	LipMag	-0.11	0.28330287
82	FlowerArea	LipYel	-0.12	0.27234977
83	PetalArea	LipYel	-0.12	0.25240006
84	LipW	PetalMag	-0.13	0.23945199
85	SepalL	LipYel	-0.13	0.21883355
86	LipArea	PetalMag	-0.15	0.15710648
87	LipL	PetalMag	-0.15	0.14957479
88	PetalL	LipYel	-0.16	0.13557035
89	LipArea	LipMag	-0.16	0.12435131
90	LipL	PetalMagArea	-0.18	0.09644586
91	SepalW	PetalMag	-0.19	0.07817008
92	FlowerL	LipYel	-0.19	0.07660318
93	LipW	PetalMagArea	-0.22	0.04021229

94	LipArea	PetalMagArea	-0.22	0.03500814
95	SepalArea	PetalMag	-0.23	0.02671206
96	PetalW	PetalMag	-0.24	0.0227038
97	SepalW	PetalMagArea	-0.25	0.01873125
98	SepalL	LipMag	-0.25	0.01659447
99	SepalArea	PetalMagArea	-0.26	0.01565296
100	FlowerL	PetalMag	-0.26	0.01523938
101	SepalL	PetalMag	-0.26	0.01415203
102	FlowerArea	PetalMag	-0.26	0.01398974
103	PetalArea	PetalMagArea	-0.26	0.01313921
104	PetalW	PetalMagArea	-0.26	0.01271553
105	SepalL	PetalMagArea	-0.26	0.01268041
106	SepalW	LipMag	-0.27	0.01149084
107	FlowerW	PetalMag	-0.27	0.01093822
108	PetalW	LipMag	-0.27	0.00992254
109	FlowerArea	PetalMagArea	-0.27	0.0091033
110	PetalArea	PetalMag	-0.28	0.00860927
111	PetalL	PetalMag	-0.28	0.00803583
112	FlowerL	PetalMagArea	-0.29	0.00620043
113	SepalArea	LipMag	-0.29	0.00597825
114	FlowerW	PetalMagArea	-0.32	0.00262037
115	PetalL	LipMag	-0.32	0.00196889
116	PetalL	PetalMagArea	-0.33	0.00144003
117	FlowerW	LipMag	-0.35	0.00088484
118	FlowerL	LipMag	-0.37	0.0003806
119	FlowerArea	LipMag	-0.38	0.00023445
120	PetalArea	LipMag	-0.38	0.00019548

Supplementary Table S2. Mapping rate of SSR and SNP markers in linkage analysis of F_1 population from the cross between *P. aphrodite* and *P. equestris*

Markers	Number of markers		
	SSRs	SNPs	Total
Applicable markers for linkage analysis	108	1,633	1,741
Mapper markers on the linkage map	23	1,191	1,214
Mapping rate of markers (%)[a]	21.3%	72.9%	69.7%

[a](number of mapped markers on the linkage map / number of applicable markers for linkage analysis)*100%

Supplementary Table S3. Summary of the genetic linkage map consisted of 27 linkage groups (LGs) that constructed from the F_1 population of a cross between *P. aphrodite* and *P. equestris*.

LG	Number of mapped markers			Length (cM)	Marker density (cM)[a]
	SSRs	SNPs	Total		
1	1	125	126	1979.33	15.71
2	3	141	144	1719.35	11.94
3	0	84	84	1127.93	13.43
4	0	73	73	1005.06	13.77
5	0	64	64	900.78	14.07
6	0	69	69	898.31	13.02
7	2	53	55	895.73	16.29
8	2	62	64	817.56	12.77
9	0	84	84	759.72	9.04
10	0	43	43	547.06	12.72
11	0	40	40	410.99	10.27
12	3	24	27	400.41	14.83
13	2	24	26	398.48	15.33
14	2	40	42	330.13	7.86
15	2	18	20	320.62	16.03
16	1	26	27	303.6	11.24
17	0	30	30	287.62	9.59
18	0	19	19	257.13	13.53
19	1	23	24	242.89	10.12
20	0	24	24	241.89	10.08
21	1	23	24	238.38	9.93
22	0	15	15	234.75	15.65
23	1	17	18	184.96	10.28

24	1	17	18	181.82	10.10
25	0	21	21	174.41	8.31
26	0	17	17	168.2	9.89
27	1	15	16	164.94	10.31
Total	23	1191	1214	15192.05	12.51

[a](Length of each LGs (cM) / total number of mapped markers on each LGs)

Supplementary Table S4. Summary of 19 homologous groups (HGs) that arranged from 27 linkage groups (LGs) which constructed from the F_1 population of a cross between *P. aphrodite* and *P. equestris*.

HG	No. LG contained	Number of mapped markers			Total length (cM)	Scaffold group	Total length (kb)
		SSRs	SNPs	Total			
1	6, 14	2	109	111	1,228.44	1	94,732
2	5, 12	3	88	91	1,301.19	2	82,477
3	3	0	84	84	1,127.93	3	80,517
4	1	1	125	126	1,979.33	4	68,806
5	7, 15	4	71	75	1,216.35	5	65,446
6	8, 13	4	86	90	1,216.04	6	56,085
7	20	0	24	24	241.89	7	52,232
8	18	0	19	19	257.13	8	49,399
9	10, 21	1	66	67	785.44	9	39,942
10	23	1	17	18	184.96	10	35,571
11	11, 24	1	57	58	592.81	11	32,088
12	19	1	23	24	242.89	12	30,943
13	17, 27	1	45	46	452.56	13	30,689
14	2	3	141	144	1,719.35	14	28,997
15	16	1	26	27	303.60	15	28,500
16	9	0	84	84	759.72	16	27,263
17	25, 26	0	38	38	342.61	17	24,659
18	22	0	15	15	234.75	18	24,282
19	4	0	73	73	1,005.06	19	22,873
Total	-	23	1,191	1,214	15,192.05	-	875,501
